# Evaluating fibre orientation dispersion in white matter: Comparison of diffusion MRI, histology and polarized light imaging

**DOI:** 10.1016/j.neuroimage.2017.06.001

**Published:** 2017-08-15

**Authors:** Jeroen Mollink, Michiel Kleinnijenhuis, Anne-Marie van Cappellen van Walsum, Stamatios N. Sotiropoulos, Michiel Cottaar, Christopher Mirfin, Mattias P. Heinrich, Mark Jenkinson, Menuka Pallebage-Gamarallage, Olaf Ansorge, Saad Jbabdi, Karla L. Miller

**Affiliations:** a FMRIB Centre, Nuffield Department of Clinical Neurosciences, University of Oxford, Oxford, United Kingdom; b Department of Anatomy, Donders Institute for Brain, Cognition and Behaviour, Radboud University Medical Center, Nijmegen, The Netherlands; c Sir Peter Mansfield Imaging Centre, School of Medicine, University of Nottingham, Nottingham, United Kingdom; d Sir Peter Mansfield Imaging Centre, School of Physics and Astronomy, University of Nottingham, Nottingham, United Kingdom; e Institute of Medical Informatics, Universität zu Lübeck, Lübeck, Germany; f Neuropathology, Nuffield Department of Clinical Neurosciences, University of Oxford, Oxford, United Kingdom

**Keywords:** Dispersion, Diffusion MRI, Post-mortem, Polarized light imaging, Myelin, Astrocytes, Validation

## Abstract

Diffusion MRI is an exquisitely sensitive probe of tissue microstructure, and is currently the only non-invasive measure of the brain's fibre architecture. As this technique becomes more sophisticated and microstructurally informative, there is increasing value in comparing diffusion MRI with microscopic imaging in the same tissue samples. This study compared estimates of fibre orientation dispersion in white matter derived from diffusion MRI to reference measures of dispersion obtained from polarized light imaging and histology.

Three post-mortem brain specimens were scanned with diffusion MRI and analyzed with a two-compartment dispersion model. The specimens were then sectioned for microscopy, including polarized light imaging estimates of fibre orientation and histological quantitative estimates of myelin and astrocytes. Dispersion estimates were correlated on region – and voxel-wise levels in the corpus callosum, the centrum semiovale and the corticospinal tract.

The region-wise analysis yielded correlation coefficients of r = 0.79 for the diffusion MRI and histology comparison, while r = 0.60 was reported for the comparison with polarized light imaging. In the corpus callosum, we observed a pattern of higher dispersion at the midline compared to its lateral aspects. This pattern was present in all modalities and the dispersion profiles from microscopy and diffusion MRI were highly correlated. The astrocytes appeared to have minor contribution to dispersion observed with diffusion MRI.

These results demonstrate that fibre orientation dispersion estimates from diffusion MRI represents the tissue architecture well. Dispersion models might be improved by more faithfully incorporating an informed mapping based on microscopy data.

## Introduction

By measuring diffusive motion of water molecules in tissue non-invasively, diffusion Magnetic Resonance Imaging (dMRI) aims to unravel tissue features at a much smaller scale than the imaging resolution. Obstruction of diffusion due to the presence of cellular membranes and macromolecules allows us to infer the microstructural tissue architecture that is reflected by the diffusion signal (Beaulieu, 2002). In addition to estimating microstructural tissue properties, a key challenge in dMRI is to recover within-voxel fibre configurations. Methods that have been developed to enable the reconstruction of crossing fibres in the brain are relatively well established (Behrens et al., 2003, Ozarslan et al., 2006, Tournier et al., 2007, Tuch, 2004, Wedeen et al., 2005), especially if the crossings have a high separation angle. More recently, models have been developed for specifically assessing fibre orientation dispersion using dMRI (Sotiropoulos et al., 2012, Tariq et al., 2016, Zhang et al., 2012) or dMR spectroscopy (Ronen et al., 2014). Others have focussed on the effect of dispersing geometries on the diffusion signal through Monte Carlo simulations (Kleinnijenhuis et al., 2015, Nilsson et al., 2012). Estimating dispersion has the potential to improve current tractography algorithms for delineating white matter pathways (Behrens and Jbabdi, 2009, Rowe et al., 2013) or serve as a marker of local fibre coherence, which may provide novel markers of neuropathology. In addition, the diffusion MRI signal from a large portion of white matter is better explained by a model that incorporates dispersion (Ghosh et al., 2016) than models of crossing fibres (Jeurissen et al., 2013).

Comparison of estimates against reference measurements is an essential contribution to the development of increasingly advanced models of fibre architecture. One approach is to use simulations (Balls and Frank, 2009, Hall and Alexander, 2009) or physical phantoms (Fieremans et al., 2008) to generate dMRI data that mimic those obtained from real biological tissue. The primary advantage of such an approach is the control over the fibre configuration to be investigated. A different approach is to directly compare dMRI data to a reference measure in the same tissue, for example by acquiring post-mortem MRI data and microscopy in the same tissue sample. Most commonly, the tissue is stained to highlight specific features of interest, from which quantitative measures can be derived relating to the parameters generated by the dMRI model, for example, when tissue is stained for neurites to estimate intra-cellular volume fractions of white matter. Regarding fibre architecture, many studies focus on evaluating primary fibre orientations, for example using Fourier analysis (Budde et al., 2011, Choe et al., 2012) or structure tensor analysis (Budde and Frank, 2012a, Seehaus et al., 2015). The latter was recently applied to 3D confocal microscopy in order to estimate 3D fibre orientation distribution functions (fODF) and compare them to those reconstructed from dMRI data (Schilling et al., 2016). While dispersion has been quantified previously in histological sections, for example in (Budde and Annese, 2013), a direct comparison with dMRI, ideally in the same specimens, is lacking to date.

Scanning post-mortem tissue faces several challenges compared to in-vivo dMRI experiments. For example, the apparent diffusion coefficient (ADC) and the fractional anisotropy (FA) are known to reduce in formalin fixed tissue (D’Arceuil and de Crespigny, 2007). In addition, the T_2_ relaxation time of fixed tissue is decreased compared to brain tissue of living subjects (Pfefferbaum et al., 2004, Shepherd et al., 2009). However, dMRI data with high SNR can be obtained from post-mortem tissue, because such experiments are less restricted by scan times and can be performed at systems operating at ultra-high field strengths.

In this study, we evaluated estimates of fibre orientation dispersion in white matter from post-mortem human brain specimens using a parametric dMRI dispersion model (Sotiropoulos et al., 2012) and equivalent measures derived from microscopy data. Specifically, we use polarized light imaging (PLI) measures of fibre orientation and immunohistochemical stains for myelin and astrocytes. We demonstrate good agreement between dMRI estimates of fibre orientation and equivalent measures derived from microscopy in the same three tissue samples.

## Methods

### Tissue specimens

Three post-mortem human brains were acquired with permission from the Oxford Brain Bank at the John Radcliffe Hospital in Headington, United Kingdom. The brains were immersion-fixed with formalin after extraction from the skull. Details on the history of each specimen can be found in Table 1. At the level of the anterior commissure, coronal slabs of 5 mm thickness were excised that included the medial part of the corpus callosum (CC), the centrum semiovale (CSO), part of the corticospinal tract (CST), the cingulate cortex and the superior frontal cortex. The samples originated from the anterior body of the CC, i.e. regions 3 and 4 according to Witelson's parcellation scheme (Witelson, 1989).

**Table 1:** Post-mortem specimen details. Abbreviations: PMI; post-mortem interval, i.e. time between death and start of fixation, FT; fixation time, i.e. the time between start of fixation and MRI, COD; cause of death.

#	PMI (hours)	FT (months)	Sex	Age (years)	COD
1	48	22	M	65	Myocardial Infarction
2	48	30	M	51	Chronic Obstructive Pulmonary Disease
3	16	7.5	M	91	Heart failure

Formalin fixation is known to reduce the T_2_ relaxation time of tissue, which is detrimental to SNR in MRI, but can be reversed by soaking samples in saline (Shepherd et al., 2009). The samples were immersed in phosphate buffered saline to remove excess formalin 72 h prior to imaging. 48 h later the samples were transferred to a syringe filled with Fluorinert (FC-3283, 3 M™, St. Paul, USA), a hydrogen-free liquid that is susceptibility-matched to tissue to maximize field homogeneity, but which contributes no signal. Where necessary, the specimens were immobilized by placing additional gauzes inside the syringe.

### MRI acquisition

**Fig. 1:**
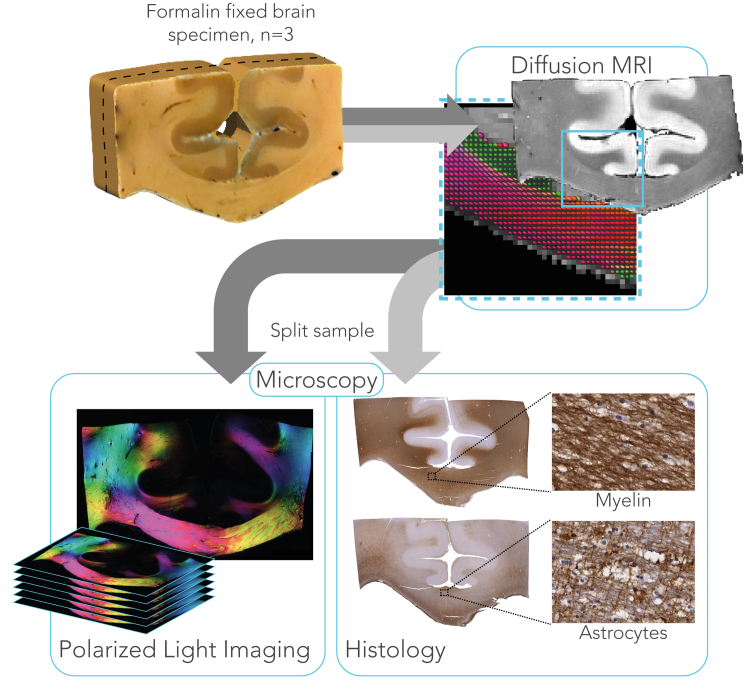
Imaging pipeline for three formalin fixed brain specimens to study fibre orientation dispersion. dMRI data were collected with 120 directions at 9.4 T, to which a parametric dispersion model was fit. After scanning, samples were cut coronally into two slabs that were processed separately to obtain fibre orientations at microscopic level. For one slab, PLI data were collected from serial sections, spanning ~1 mm in the cutting direction. The other part of the sample was immunohistochemically stained for proteo-lipid protein (PLP) as a measure of myelin content and for glial fibrillary acidic protein (GFAP) as a measure for astrocytes.

The imaging pipeline for the specimens is illustrated in Fig. 1. MR imaging was performed on a 9.4 T 160 mm horizontal bore VNMRS preclinical MRI system equipped with a 100 mm bore gradient insert (Varian Inc, CA, USA). The maximum gradient strength was 400 mT/m with a slew rate of 2162 mT/m/ms in all axes. RF transmission and reception was performed using a 26 mm ID quadrature birdcage coil (Rapid Biomedical GmbH, Germany). Diffusion-weighted images were acquired with a spin-echo sequence (TE = 29 ms, TR = 2.4 s) using single line readout and b = 5000 s/mm^2^ (δ = 6 ms and Δ = 16 ms). High b-values were required to obtain sufficient diffusion contrast for estimating dispersion, as demonstrated in (Sotiropoulos et al., 2012) using b-values as high as b = 8000 s/mm2 in post-mortem macaque brain. A total of 120 gradient directions were acquired in addition to four images with negligible diffusion weighting (b ≈ 8 s/mm^2^). The field-of-view covered the samples in the sagittal plane of the scanner (51.2 mm × 38.4 mm) and was sampled with a 128 × 96 matrix. This lead to an in-plane resolution of 0.4 × 0.4 mm, which was matched with a slice thickness of 0.4 mm for isotropic voxels. The average SNR for the b = 5000 s/mm^2^ data was 15.5 and 18.6 for grey and white matter, respectively. To reduce Gibbs ringing, the complex k-space data of all volumes were filtered with a Tukey window (α = 0.5). Diffusion tensor images (DTI) were obtained using FMRIB's Diffusion Toolbox (FDT) in FSL (Jenkinson et al., 2012) to compute mean diffusivity maps. These maps were solely used image registration with PLI and histology data. However, no diffusivity values were derived from the DTI analysis.

### dMRI-derived dispersion

**Fig. 2:**
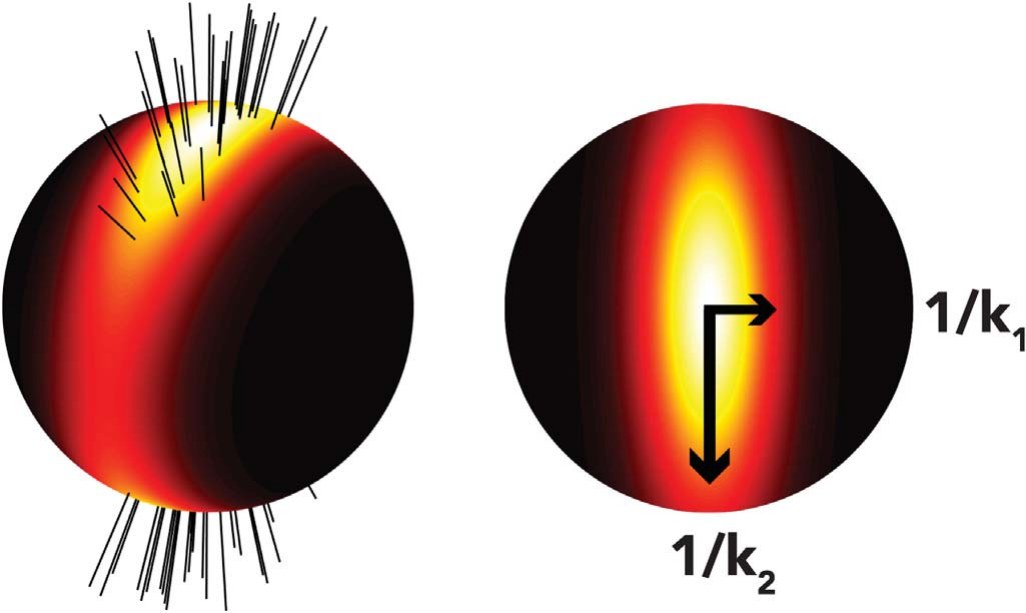
Probability density function of the Bingham distribution. The left image depicts a fibre population presenting anisotropic dispersion to which a Bingham distribution is fitted. From the Bingham distribution on the right, concentration parameters k_1_ and k_2_ can be extracted which are reciprocally related to dispersion.

The dMRI dispersion model separates the diffusion signal into isotropic and anisotropic fractions. Dispersion is estimated from the anisotropic fraction, which aims to describe both intra- and extracellular compartments. The isotropic fraction likely captures both free water and non-neuronal cells (Azevedo et al., 2009). This model describes the fODF using a Bingham distribution (Fig. 2), which provides a quantitative estimate of fibre dispersion representing the fanning and bending fibre geometries that appear throughout the brain (Sotiropoulos et al., 2012, Tariq et al., 2016). The Bingham distribution (spherical version of a Gaussian distribution) is parametrised with a 3 × 3 rank-2 matrix whose non-zero eigenvalues k_1_ and k_2_ quantify dispersion along two orthogonal axes, with low k corresponding to high dispersion. For anisotropic dispersion, k_1_>k_2_, while for isotropic dispersion k_1_ = k_2_ = κ (equivalent to the Watson distribution). A brief overview of the formulation of this dispersion model is given in Appendix A, with further details and discussion of its implementation given elsewhere (Sotiropoulos et al., 2012).

In keeping with previous work (Tariq et al., 2016, Zhang et al., 2012), an Orientation Dispersion Index (ODI) was defined. Here, we quantify the ODI in a 2D plane, because the fibre orientations derived from the microscopy data were restricted to 2D. A mathematical description to derive 2D dispersion is given in Appendix B. The eigenvalue, k_2D_, that quantifies dispersion in a plane parallel to the microscopy data was used to compute the ODI as in Eq. (1). An ODI of 0 corresponds to perfectly aligned within-voxel fibre orientations (no dispersion), while an ODI of 1 represents a uniform distribution of fibres on a circle.

(1) $$\mathrm{ODI}=\frac{2}{\pi }\arctan(1/k_{2D})$$

In addition to the dispersion model, fODFs were obtained. The fODFs were solely collected to compare the alignment of fibre orientation between dMRI and microscopy-derived fibre orientation distributions (FOD) after co-registration, but no dispersion estimates were derived from the fODFs. The fODFs were obtained via Constrained Spherical Deconvolution (CSD). Spherical harmonics coefficients up to the 8th order were fitted in MRtrix (Tournier et al., 2013). Because the microscopy techniques yield fibre orientation in 2D, the fODF of each voxel was projected into a 2D plane. A convex hull was calculated from the 2D projection and referred to as fODF_2D_ hereafter.

### Microscopy

Estimates of dMRI dispersion were compared to two microscopic imaging approaches: polarized light imaging (PLI) and immunhistochemical staining. After MRI, each sample was sliced along the middle, in the coronal plane, to generate two slabs of approximately equal thickness (Fig. 1). One of the slabs was frozen for PLI and the other was processed and paraffin embedded for histology. Frozen tissues are ideal for PLI as lipids in the myelin sheath are preserved. Fibre orientation distributions were derived from both PLI and histology using structure tensor analysis, as described below.

#### Polarized light imaging

PLI enables quantification of fibre orientations at microscopic resolution based on the optical property of birefringence (Axer et al., 2011a, Larsen et al., 2007). The myelin sheath surrounding axons is birefringent de Campos Vidal et al., 1980), and results in a transmitted light intensity that depends on the angle between the myelin sheath and a polarizing filter on the microscope. Fibre orientation estimates are generated in PLI by acquiring a series of images with varying polarizer angle. The resulting light intensity exhibits a sinusoidal variation, the phase of which indicates in-plane fibre orientation.

For PLI, the tissue slabs were immersed in a 30% sucrose-solution for one week to prevent the formation of ice crystals during freezing and to preserve tissue morphology. After cryoprotection, the tissues were frozen to −80 °C. One day later the tissue slabs were sectioned at −12 °C in the coronal plane using a cryostat (Leica, Germany), at a thickness of 60 µm. Sixteen sections were collected in a serial manner for each specimen, covering a distance of ~1 mm in the cutting direction. Images were taken on a Leica DM4000B microscope (Leica, Germany) equipped with a polarizer, a rotatable polarizer (the analyser), a quarter wave plate (QWP) and a white LED light source. The fast axis of the QWP was oriented 45° with respect to the transmission axis of the polarizer to create circularly polarized light. The rotating analyser then captured the phase shift induced by the myelin sheath. Images were taken at 18 equiangular rotations ranging from 0° to 170° with a 1.25x magnifying objective yielding a resolution of 4 µm/pix. Background correction of the images was performed as described elsewhere (Dammers et al., 2010). Fibre orientations were derived by fitting the light intensity at each pixel to a sinusoid, the phase of which gives the in-plane orientation (Axer et al., 2011b) (see Appendix C). A histogram of the fibre orientations from a local neighbourhood of 100 × 100 pixels across 6 slices (i.e. 0.4 × 0.4 × 0.36 mm) was computed to obtain a fibre orientation distribution (FOD).

#### Histology

After embedding in paraffin, samples were cut into 6 µm thick sections for immunohistochemistry. The tissue sections were stained with antibodies against myelin proteo-lipid protein (PLP; MCA839G; Bio-Rad; 1:1000) or glial fibrillary acidic protein (GFAP; Z0334; DAKO; 1:2000) for astrocytes and visualised using DAKO REAL™ EnVision™ Detection System (K5007) with diaminobenzidine (DAB) chromogen. Sections were counterstained with haematoxylin. For every specimen, six sections were stained, three for each protein. Digitization of the stained sections was performed on a Leica Aperio Scanscope AT Turbo slidescanner (Leica, Germany) using x40 magnifying objective, leading to a resolution of 0.28 µm/pix.

#### Structure tensor analysis

**Fig. 3:**
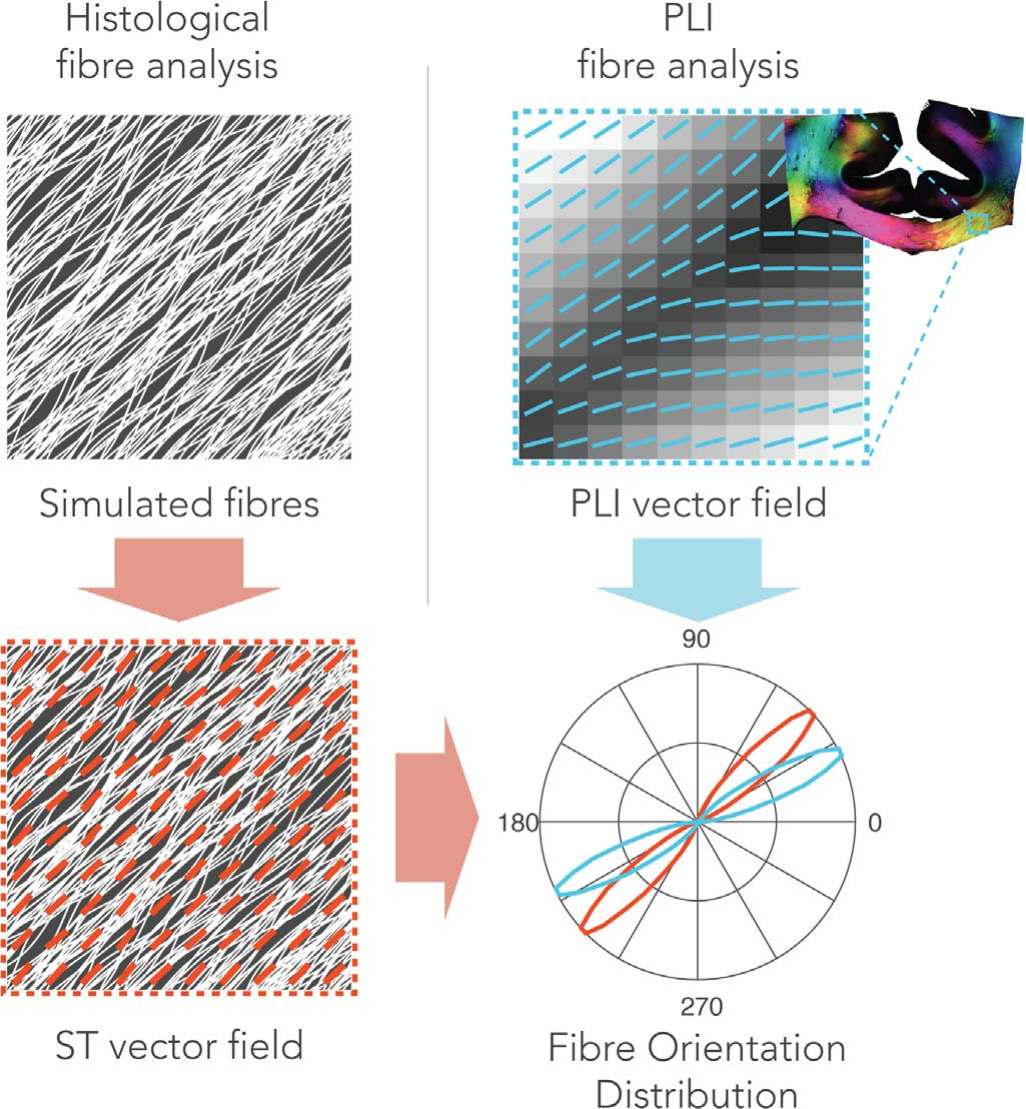
Microscopic fibre orientation analysis for histological and PLI data. Structure tensor analysis was performed on histology (left column). Here, a simulated fibre configuration is shown for demonstration purposes. Fibre orientation at each pixel was derived by estimating the direction of local intensity gradients. An FOD (red) can then be calculated by taking the histogram of fibre orientations in a neighbourhood comparable to the size of an MRI-voxel. For PLI data, a similar FOD can be obtained (blue). Instead of analysing texture in an image, PLI yields a fibre orientation at each pixel after PLI-signal processing. Like the structure tensor analysis, an FOD was extracted by computing the histogram of orientations from the PLI vector field in a local neighbourhood.

**Fig S1:**
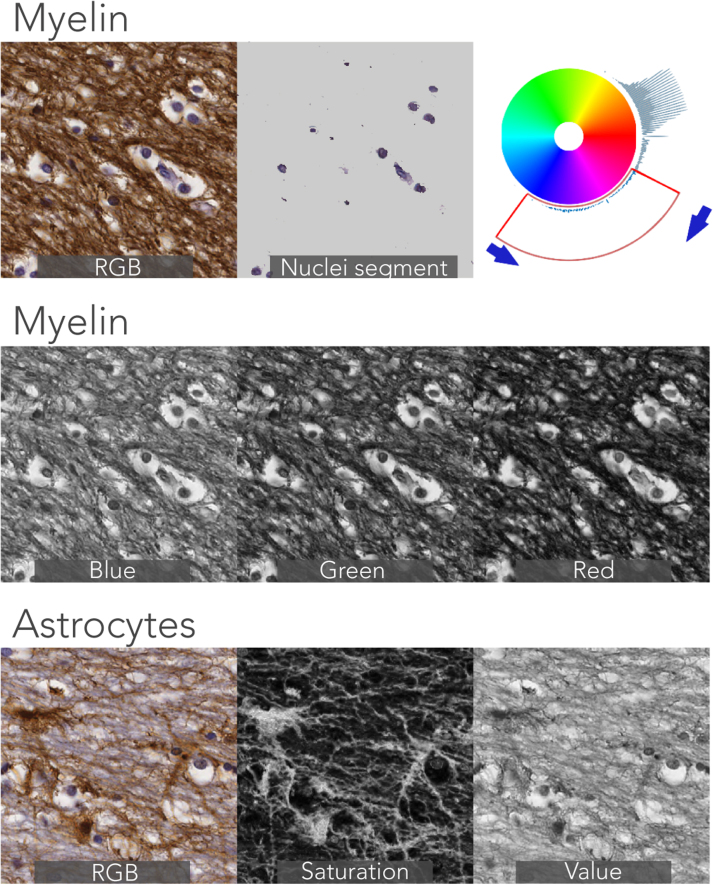
Pre-processing of the histological images. **Top row:** Based on an empirically defined RGB-threshold, cell-nuclei were removed from both the myelin images and the astrocyte images (the latter not shown here). **Middle row:** The colour-channel with the strongest staining intensity was selected for further processing, the red-channel in our case. **Bottom row:** To enhance astrocyte processes, the images were transformed to HSV-colourspace. The saturation-channel of the HSV images appeared to have contribution from primarily astrocyte staining and was used for further processing..

Fibre orientations in the histological images were obtained via texture analysis. The histological FODs were derived after structure tensor analysis of the images (Bigun and Granlund, 1987). Images were first subdivided into patches of 1400 × 1400 pixels, roughly matching the dimensions of an MRI-voxel in our experiment. From the myelin images, the red colour-channel was used for analysis. The astrocyte images were first transformed to the HSV colourspace. The saturation channel was then extracted as it was mostly represented by astrocyte staining without much contribution from the background staining. For both stains, the cell-nuclei were removed from the images using colour-based segmentation which was facilitated by their blue appearance. To do so, an empirically defined RGB threshold was used to define a mask representing the cell-nuclei. Examples of these pre-processing steps are depicted in Supplementary Figure S.1. Structure tensor analysis was performed on each of the processed patches separately. We calculated a fibre orientation estimate at every pixel as described previously (Budde and Frank, 2012b, Rezakhaniha et al., 2012) (see Appendix D for a mathematical formulation). Fibre orientations were extracted from pixels above a staining intensity threshold to minimize the effect of background staining. The FOD was then computed as the histogram of the extracted fibre orientations. An overview of the FOD derivation can be found in Fig. 3.

#### Microscopy-derived dispersion

Analogous to the dMRI dispersion model, the Bingham distribution was fitted to the microscopy-derived FODs (Riedel, 2015). The Bingham eigenvalues were extracted to obtain microscopic orientation dispersion. However, the Bingham distribution is parameterized in 3D, whereas the microscopy FODs are in 2D. Fitting the Bingham to the 2D microscopic FODs theoretically sets k_1_ → ∞, leading to a single dispersion axis, since the FOD is a delta function in the third dimension. Hence, the eigenvalue representing the only non-zero dispersion was used to compute an orientation dispersion index (ODI) as defined in Eq. (1).

### Registration

For MRI - microscopy comparisons with spatial specificity, an essential step is the alignment of each modality into the same space. Pixel-wise comparison is even possible with sufficiently accurate alignment of the microscopy slices to the MRI data. However, tissue processing steps such as fixation, embedding and cutting cause non-linear deformations to the tissue sections, making this a much more difficult alignment than is faced in conventional MRI. Here, a 2D deformable registration was employed using a Modality Independent Neighbourhood Descriptor (MIND) (Heinrich et al., 2012). The images used in calculating alignment for each modality were: the mean diffusivity maps for MRI, the transmittance maps for PLI (i.e. I_0_ maps, see Appendix C), and the greyscale stained images for histology. The computed deformation fields were then applied to the corresponding FOD maps to be able to compare FODs from individual voxels between the modalities. Prior to transforming the FOD images, the fibre orientations were reoriented in order to preserve the fibre orientations relative to the bulk anatomy. For each point in the deformation field, a local affine transformation can be computed as described previously (Alexander et al., 2001). The rotation induced by this affine transformation was used to reorient the FODs.

### Comparison of dMRI and microscopy dispersion

#### Regions of interest

Three regions of interest (ROIs) were defined in the specimens with known increasing grades of dispersion: the corpus callosum (CC), the corticospinal tract (CST) and the centrum semiovale (CSO). The masks for the ROIs are shown in Fig. 10 for specimen 1. Our goal in including the CSO is to span the broad range of ODI values found in the brain. The CSO is a region of crossing fibres, which is an extreme form of dispersion that would ideally be described by a more complex model, such as a sum of multiple Bingham distributions. The comparison of the diffusion signal against microscopy in the CSO is therefore informative, but ODI values themselves are to be interpreted with caution. Average ODI values were extracted from each ROI and correlated against each other. For the corpus callosum a more detailed comparison was given by averaging ODI values along the superior-inferior direction, yielding left-to-right dispersion profiles which were correlated across modalities. Finally, a voxel-wise comparison of dispersion was facilitated by co-registration of the different modalities.

#### Role of astrocytes

As a secondary goal, we evaluated whether dMRI dispersion is better explained by both myelinated axons and astrocytes, compared to only myelinated axons. A combination of FODs derived from the myelin and astrocyte histology was defined as FOD_MA_:

(2) $${\mathrm{FOD}}_{\mathrm{MA}}\left(\theta \right)=f_{M}\;∙\;{\mathrm{FOD}}_{M}\left(\theta \right)+f_{A}\;∙\;{\mathrm{FOD}}_{A}(\theta )$$

**Fig S.3:**
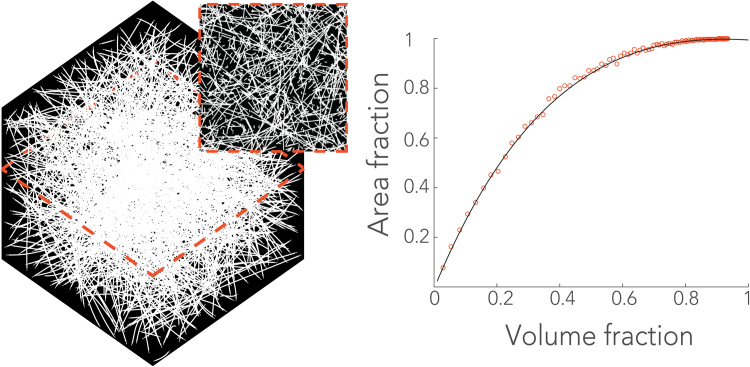
Simulation to estimate the volume fraction of fibres using the area fraction derived from 2D microscopy images. A 3D voxel space was created in which randomly packed fibres at random orientation were added. At each increase in the fibre volume fraction, a virtual slice was taken from which an area fraction was estimated as the percentage of positive pixels after thresholding, just as in the histological images. This yielded a mapping between the area fraction and the volume fraction that is given by the curve on the right.

Here, FOD_M_ and FOD_A_ are the myelin and the astrocyte derived FODs, respectively. Likewise, f_M_ represents the volume fraction of myelin and f_A_ is the volume fraction of astrocytes. The FODs were defined along orientation angles θ. As described in section 2.4.3, fibre orientations were only extracted from pixels above a certain staining intensity after pre-processing the images. The percentage of pixels above this staining intensity was defined as the area fraction for the studied structure. From the area fraction, a volume fraction was estimated via a numerical simulation. In brief, a 3D voxel space was created in which randomly oriented fibres were generated at increasing volume fractions. For each increase in volume fraction, a virtual slice was extracted from the 3D volume. From the slice, an area fraction was estimated by segmenting the fibres and counting the number of pixels relative to the total area, just as in the histological images. In such a way, an informed mapping was constructed that relates the measured area fraction to a volume fraction (see Fig. 5 and Supplementary Figure S.3). Hence, to obtain f_M_ and f_A_, the computed area fractions were converted to volume fractions based on the mapping. In addition, the volume fractions were equally scaled for each location such that f_M_ + f_A_ = 1.

## Results

### Registration

**Fig. 4:**
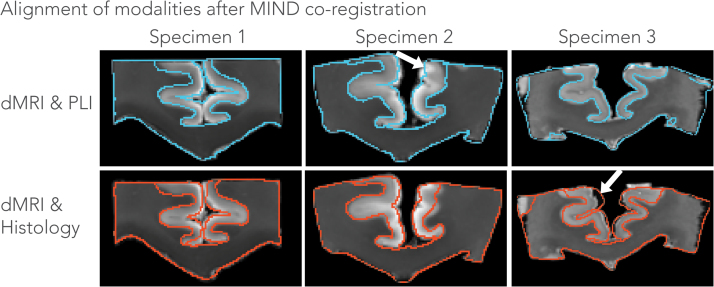
Contour plots depicting the alignment of the modalities after registration using the MIND algorithm. Shown are the mean diffusivity maps superimposed with either PLI (edges of transmittance maps in blue) or histology (edges of myelin stain in red) for each specimen. In most areas, the alignment is highly accurate (within the size of a voxel, i.e. 0.4 mm), while the white arrows indicate regions where the registration did not overlap the corresponding modalities.

Fig. 4 depicts the alignment of all three modalities, i.e. mean diffusivity maps (dMRI), transmittance maps (PLI) and the greyscale stains (histology) using co-registration with the MIND algorithm. The approach provided good alignment of the images by computing a local similarity metric rather than a global similarity metric such as mutual information that is used in many common registration frameworks for biomedical imaging. The local similarity metric is modality independent and was found to be well suited for our MRI – microscopy comparisons. Judging from the close correspondence between the boundaries (within the size of a voxel, i.e. 0.4 mm) of the CC, alignment of the different modalities appears to have reached good accuracy despite the severe deformations commonly associated with tissue processing for microscopy. However, some misalignment is also visible in some cortical regions, for example between dMRI and histology in specimen 3 indicated by the white arrow. As a result, we expect voxel-wise comparisons to be more robust in the CC than in some of the other regions contained in these samples.

### FOD microscopy

**Fig. 5:**
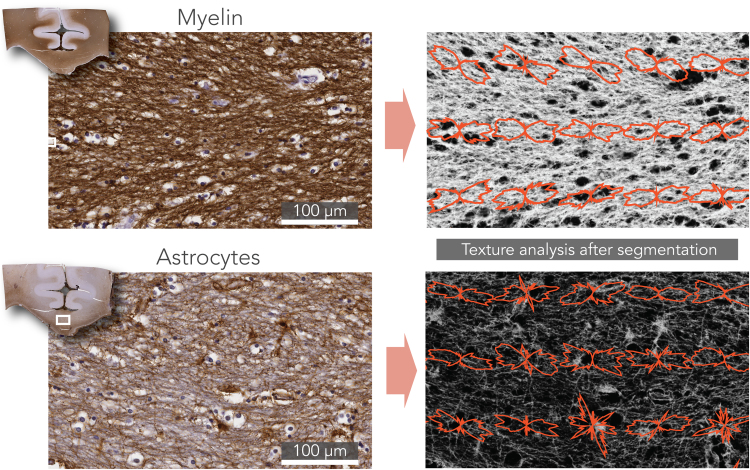
Fibre orientation analysis in histology. Structure tensor analysis was performed to obtain a fibre orientation at each pixel. The left panels show the original images of two field-of-views depicted from the corpus callosum (white boxes). In both cases, the stain of interest is brown and blue/purple is a counterstain that depicts cell nuclei. To highlight the feature of interest (i.e. myelin or astrocytes), the images were first pre-processed as shown in the right panels. Next, structure tensor analysis was performed on the processed images. Fibre orientations were extracted only from pixels above a certain staining intensity. For illustration purposes, the neighbourhood over which an FOD is computed here is smaller than the size of an MRI-voxel in our experiment.

The PLI and histology images allow the relative contribution of distinct sources of dispersion to be distinguished. To achieve this, FODs were obtained from the myelin (FOD_M_) and the astrocyte (FOD_A_) histology data and combined (Eq. (2)) to yield the aggregate FOD_MA_. Fig. 5 depicts two regions in the corpus callosum that illustrate these two sources of dispersion. The images were first pre-processed to highlight the feature of interest, as described above. Structure tensor analysis was performed to obtain a fibre orientation at every pixel. In the myelin image, fibre populations oriented at large angles (~40 degrees) to one another are running in close proximity within a relatively small field-of-view. In general, the astrocyte processes follow the orientation of the highly anisotropic matrix of axons in which they are embedded, though in a less ordered manner, yielding wider FODs and thus higher dispersion.

**Fig. 6:**
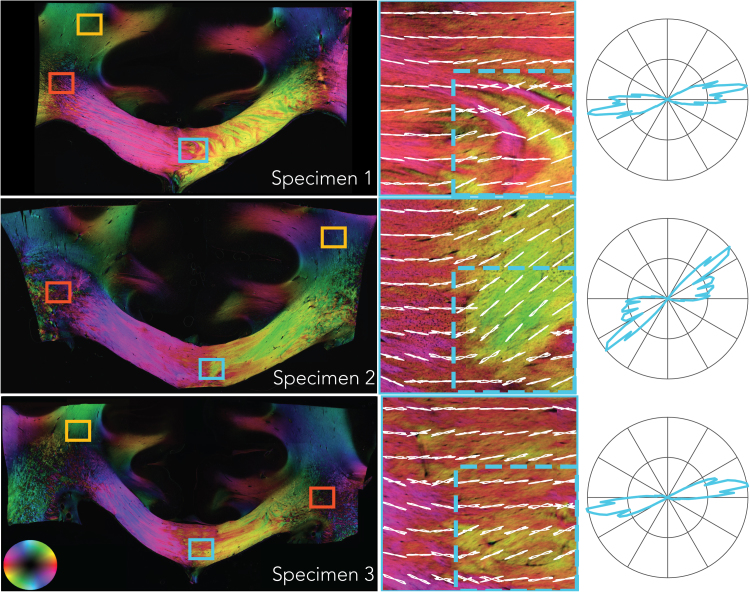
PLI fibre orientation maps of each specimen. In-plane orientation is colour-coded according to the sphere on the bottom left. CC regions of interest in the middle row are taken from the blue boxes in the images (left column). The red and yellow boxes indicate areas in the centrum semiovale and the corticospinal tract, respectively and are given in Fig. 7. FODs from a neighbourhood size of 0.4 × 0.4 mm (white lines) are superimposed on the high-resolution fibre orientation maps (shown colour-coded). The dashed boxes illustrate a neighbourhood corresponding to a typical dMRI voxel for in-vivo experiments (2 mm). FODs from these dashed boxes are depicted in the polar plots on the right and exhibit greater dispersion than the smaller neighbourhoods.

A representative slice of the PLI-derived fibre orientation maps is given in Fig. 6 for each specimen, with colour representing orientation (note that the colour-map only codes the in-plane orientation and therefore differs from those typically used in dMRI, as shown in the lower left-hand corner). At the mesoscopic scale, the corpus callosum exhibits a considerable amount of heterogeneity of fibre orientation. A loss of coherence is evident at the midline, where small fascicles appear to intertwine and change direction. FODs at the scale of the MRI voxels in our experiment (0.4 mm) are shown for a 3.2 × 3.2 mm region at the midline of the corpus callosum (middle column). When computing the FODs at the scale the size of a voxel for a typical in-vivo dMRI experiment (2 mm; dashed region) dispersion increases, as can be seen in the polar plots (right column).

**Fig. 7:**
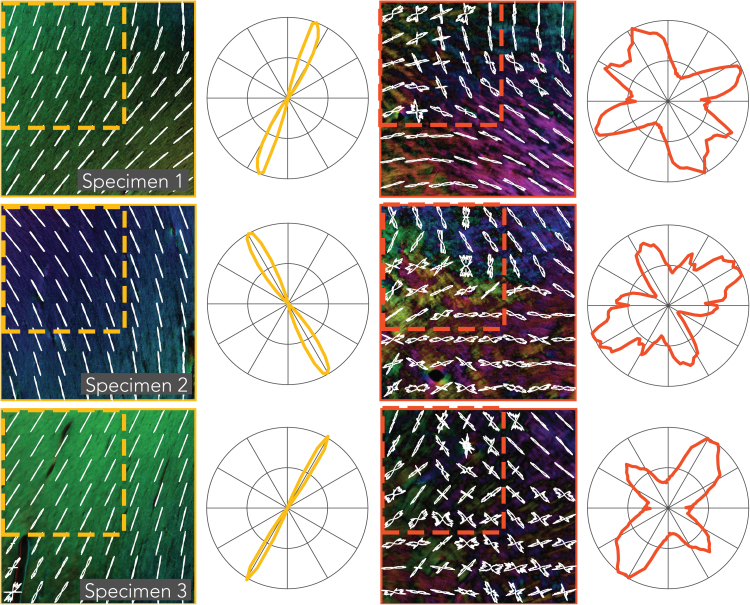
PLI fibre orientation maps for the corticospinal tract (yellow) and the centrum semiovale (red). See Fig. 6 for further description.

Fig. 7 depicts the PLI FODs for the CST and the CSO from locations indicated by the yellow and red boxes in Fig. 6, respectively. The CST has been established to exhibit higher dispersion than CC; this is not evident in the PLI images, which is likely due to sectioning of the tissue samples in the coronal orientation, whereas the major axis of dispersion in the CST is along the anterior-posterior direction. The crossing of fibres from the CC and CST in the CSO are clearly visible as these run in the coronal plane. A third bundle crosses in the CSO, the superior longitudinal fasciculus, which runs into the imaging plane; fibres perpendicular to the section cannot be estimated using our PLI setup and therefore these fibres are likely the source of the black speckling in the CSO.

**Fig. 8:**
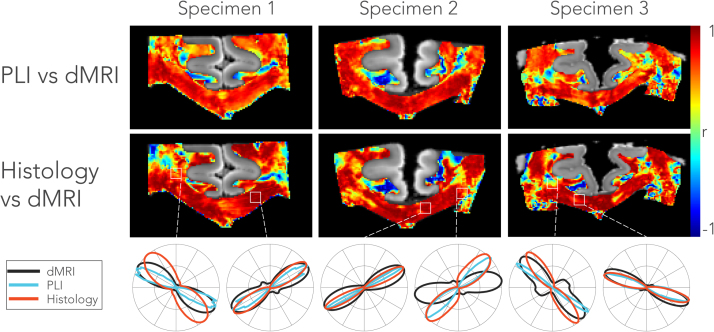
Correlation of microscopy derived FOD and fODF_2D_ amongst modalities in each of the three specimens. The top row depicts the correlation coefficients between the FOD from a stack of 6 PLI slices (dimensions: 0.4 × 0.4 × 0.36 mm) and the fODF_2D_ from the corresponding MRI slice (0.4 mm isotropic voxels). The histological FODs were obtained from the average of three myelin and three astrocytes stains combined (i.e. FOD_MA_). The bottom row depicts the FODs from each modality for a voxel that is representative for the region of interest marked with the white rectangles above.

To evaluate the overall correspondence of the FODs calculated from the different modalities within each 0.4 × 0.4 mm neighbourhood, we calculated the correlation coefficients between amplitudes (in a polar coordinate system) of the microscopy-derived FODs and dMRI-derived fODFs_2D_ (Fig. 8). FODs in the CC are in general highly correlated, while regions such as the CSO or the cingulum exhibit weaker, or even negative, correlations. The discrepancy in these more complex fibre geometries and fibres perpendicular to the image plane are in part due to projecting 3D fODFs onto the 2D plane. Poor agreement in the cingulum is particularly to be expected given that these fibres run approximately into the plane of sectioning. The maps in Fig. 8 indicate both that the FODs and fODFs_2D_ are generally in agreement (with the correlation coefficients likely dominated by directional similarity) and that spatial alignment is of sufficient quality to attempt comparisons between the techniques at the voxel (pixel) level.

### Dispersion

**Fig. 9:**
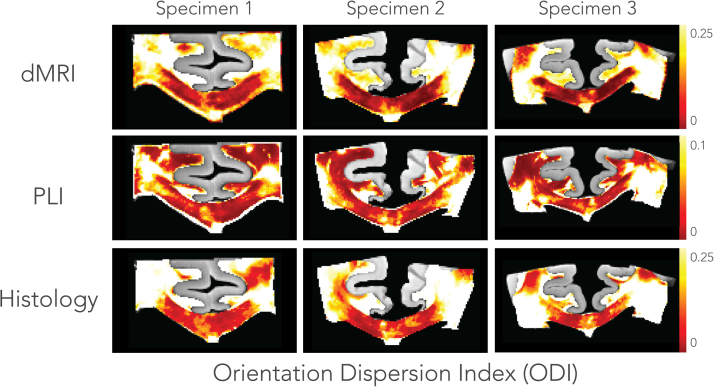
Orientation dispersion maps across specimens and modalities. Dispersion in the dMRI was estimated for each voxel by fitting a dispersion model parametrised by the Bingham distribution (Appendix A). Dispersion in the microscopy images was estimated by fitting the Bingham distribution to the FODs in a local neighbourhood comparable to the size of an MRI voxel. The histological dispersion is derived from myelin only (i.e. FOD_M_). Lower dispersion values were found in PLI (note the difference in the range of the colour bar), while dMRI and histological dispersion have similar ODI values. Focusing on the corpus callosum, an increased amount of dispersion at the midline is consistently observed across the modalities and specimens.

An overview of dispersion estimates for each specimen and modality is given in Fig. 9. The histological dispersion shown here was derived from the myelin FOD_M_. Broadly similar patterns can be recognized in the ODI maps, with high dispersion in crossing fibre regions like the CSO and lower dispersion in the CC. However, on a voxel-wise level, not all regions across the modalities show a consistent pattern of dispersion. In terms of absolute values, dMRI and histology exhibit similar dispersion values, while dispersion from PLI is considerably lower. Focusing on the CC, the midline exhibits more dispersion as compared to its lateral aspects in all modalities. The highest dispersion is found in the CSO due to the crossing of the CC, the CST and superior longitudinal fasciculus. However, we hypothesized that dispersion in the CST is lower than in the CSO, but for dMRI the difference appears to be little.

**Fig. 10:**
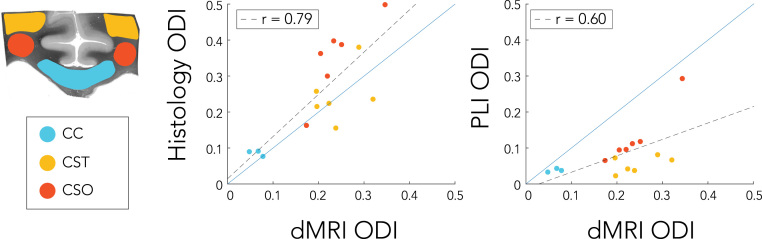
ROI analysis of dispersion estimates (ODI) in the corpus callosum (CC), the corticospinal tract (CST) and the centrum semiovale (CSO). The dots represent different specimens and regions, with each dot is colour-coded according to the region it belongs to. A trend line (dashed black) is given for the relation between modalities in addition to the trend line indicating a one-to-one relationship (solid blue).

The region-wise analysis yielded an average ODI for each specimen across the modalities and different ROIs. Scatterplots relating dMRI dispersion to either PLI or histology (myelin) dispersion are given in Fig. 10. A higher correlation was found between dMRI and histology (r = 0.79) than between dMRI and PLI (r = 0.60). In addition, the trend line between dMRI and histological dispersion almost equals a one-to-one relationship. CC dispersion was found to be the lowest, while the CSO exhibited higher dispersion than the CST for PLI and histology. However, for dMRI the dispersion was roughly similar in the two latter regions.

**Fig. 11:**
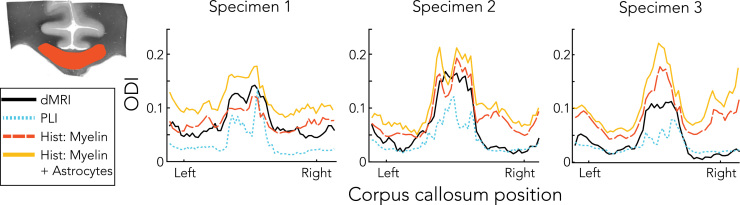
Dispersion profiles in the corpus callosum for dMRI, PLI and histology. Profiles were obtained by masking the dispersion maps with a corpus callosum mask as in the example on the top left. The mean along superior-inferior directions and across slices resulted in the dispersion profiles shown here. Similar to the pattern in the spatial maps (Fig. 9), an increased amount of dispersion is evident around the midline compared to the lateral aspects of the corpus callosum. PLI exhibits lowest dispersion in the corpus callosum, whereas histology and dMRI yield similar values. However, when astrocytes (in addition to myelin) were incorporated for the histological dispersion estimate (i.e. derived from FOD_MA_), higher dispersion values are obtained.

Fig. 11 plots the average ODI profiles for each left-to-right location of the CC. Two histology-based dispersion profiles are shown: one based purely on the myelin staining and one combining myelin and astrocyte stains based on the combined FOD_MA_ (Eq. (2)). The ODI consistently peaks in the medial part of the corpus callosum both across modalities and specimens. Dispersion derived from myelin alone better reflects the dMRI dispersion than dispersion derived from both myelin and astrocytes. These results suggest that, although astrocytes exhibit dispersion, their contribution to the shape of the dMRI profile is small.

**Fig. 12:**
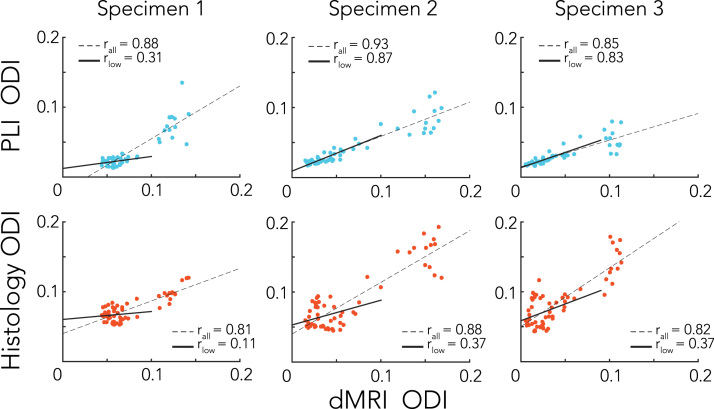
Scatter plots of the dispersion profiles extracted from the corpus callosum as in Fig. 11. The top depicted the comparison of PLI and dMRI, while the bottom row shows histology (myelin only) and dMRI. Correlation coefficients were calculated for all points together and separately for ODIs below 0.1, to see if a similar trend exists for a smaller fraction of the data. The solid trend lines indicate whether low ODIs follow a similar behaviour as the relationship of all ODI values together (dashed trend lines).

A correlation of the dispersion profiles across the CC demonstrated very similar patterns of dispersion for the different modalities, with the highest correlation coefficient reported for specimen 2 between the dMRI and PLI dispersion profiles (r = 0.93), see Fig. 12. These high correlation coefficients were to some extent driven by the low-vs-high dispersion clusters in the CC (corresponding to the medial and lateral CC). This was especially evident for specimen 1, where little correlation is found when only ODIs below 0.1 (for dMRI) were considered, reflecting the very narrow range of ODI values in the lateral CC. For specimens 2 and 3, however, the slopes of the low ODIs follow a similar trend compared to all ODI values and, in case for PLI, even with comparable correlation coefficients.

**Fig. 13:**
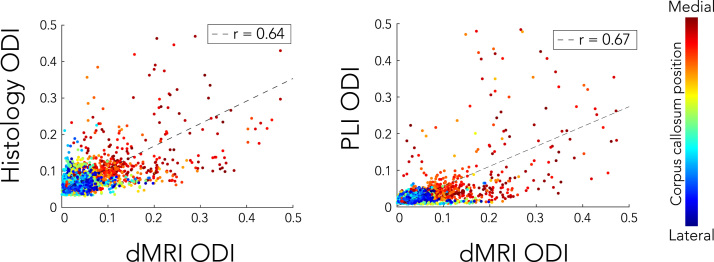
Voxel-wise correlation of dispersion estimates in the corpus callosum for PLI and histology compared to dMRI. Each dot is colour-coded according to their medial-lateral position in the corpus callosum. Notice that the outliers primarily correspond to high ODIs in the centre of the corpus callosum.

**Fig. 14:**
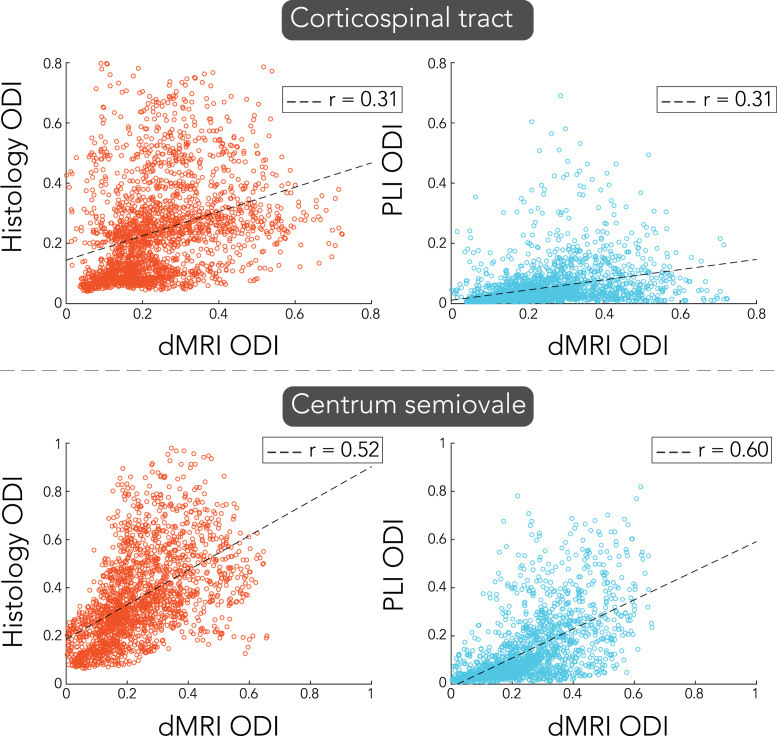
Voxel-wise comparison of orientation dispersion index (ODI) of dMRI with either histology (left) or PLI (right) in the corticospinal tract and the centrum semiovale.

Fig. 13 features a voxel-wise comparison of dispersion in the CC. Each dot is colour-coded according to its medial-lateral location across the CC. Overall, lower correlation coefficients (r = 0.64 and r = 0.67 for histology and PLI, respectively) are found as compared to the ROI analysis. The correlations are influenced by outlier voxels in the medial part of the CC, where fibre structure is chaotic (see Fig. 6). Because dispersion is more heterogeneous in the medial CC, minor misalignments could be one source of disagreement between modalities. Voxel-wise comparisons of dispersion for the CST and CSO are given in Fig. 14. While for the CSO moderate correlation (r = 0.52 and r = 0.60 for histology and PLI, respectively) was found, lowest correlation coefficients were reported for the CST (r = 0.31 for both histology and PLI).

## Discussion

This study investigated fibre orientation dispersion in white matter derived from dMRI and microscopy. Estimates of dispersion were previously reported in human brain tissue with MRI using either dMRI (Sotiropoulos et al., 2012, Tariq et al., 2016, Zhang et al., 2012) or dMRS (Ronen et al., 2014) and with structure tensor analysis of histological sections (Budde and Annese, 2013); however, an evaluation across modalities has been lacking to date. Here, we estimated dispersion from dMRI data using a two-compartment model and compared these against dispersion estimates derived from microscopy data, including PLI and histology.

The most pertinent question addressed in our study is whether dispersion estimated from dMRI is a result of actual fibre orientation dispersion or primarily determined by orthogonal diffusion due to variation in other microstructural properties like axon diameter or extracellular volume fraction. The ROI analysis yielded strong correlation between dispersion derived from dMRI and histology, and a somewhat weaker correlation for dMRI and PLI (Fig. 10). Focusing on the CC, we found very high correlation between dispersion profiles from left-to-right, and lower correlations at voxel-wise level (Fig. 12, Fig. 13). Overall this provides strong evidence that the dispersion estimates from dMRI models capture actual microstructural fibre orientation dispersion. This conclusion was substantiated using two independent measures of fibre orientation: the birefringence of the myelin sheaths using PLI and the texture of myelinated fibres visualised with proteo-lipid-protein stains for myelin.

The ROIs were selected based on their expected variation in dispersion, with the CC expressing low dispersion, the CST medium dispersion and the CSO high dispersion due to crossing fibres. However, the distinction between medium and high dispersion was not apparent for dMRI, with the CST and CSO yielding similar dispersion (Fig. 10). The strongest correlations were found in the CC (Fig. 13, Fig. 14), for which the fanning is largely in the imaging plane of microscopy. For the CST and CSO, the fibre configurations extend through the imaging plane of microscopy, which may explain the relatively worse agreement with dMRI in these regions. The CST has its primary fanning along the anterior-posterior direction, and as such, the PLI derived FODs demonstrate relatively little dispersion in the CST (Fig. 7). The CSO exhibits well-established crossings along all three dimensions, but only two of these are captured by the microscopy images. Thus, although dMRI derived dispersion was extracted along the imaging plane of microscopy, the specific anatomy for each ROI may have played a role in the correlation coefficients reported here.

The microscopy techniques employed in this study face some intrinsic limitations due to the restrictions of 2D measurements. PLI has potential to quantify the 3D fibre orientation by estimating the inclination angle (see Eq. (C.2), in Appendix C). However, with data acquired from the PLI setup used here, the inclination angle has an ambiguous solution and requires assumptions about tissue properties including the birefringence of myelin and the slice thickness. This limitation can be overcome by changing the propagation direction of the light through the specimen, either by tilting the microscope stage (Axer et al., 2011b, Wiese et al., 2014) or changing the direction of the light with micro-lens arrays (Oldenbourg, 2008). Alternatively, one could obtain reference measurements of 3D FODs using confocal microscopy with 3D structure tensor analysis (Khan et al., 2015, Schilling et al., 2016) or optical coherence tomography (Magnain et al., 2014, Srinivasan et al., 2012). The latter is also less susceptible to tissue deformations, as the image acquisition is performed en bloc prior to cutting.

Validation studies of dMRI often consider reference measurements derived from microscopy as being a gold standard. Typically, microscopy focuses on a specific feature of interest inside tissue that is related to the output of the dMRI model, for example myelinated axons and astrocytes in the present study. As such, other structures that possibly also influence the dMRI results may be overlooked. In that sense, 3D electron microscopy is perhaps the only technique to be considered as gold standard, as it is capable to quantify the full complexity of tissue microstructure at ultra-high resolution (Mikula et al., 2012). However, such experiments are limited to very small regions of interest, very laborious and requires sophisticated segmentation algorithms to identify tissue compartments. With regards to the former, PLI is a relatively novel imaging technique and just a few studies utilized it to evaluate dMRI results, though in a qualitative manner (Caspers et al., 2015, Leuze et al., 2014). Here we show for the first time a quantitative analysis of PLI and the results from this study may inform future studies considering PLI as a reference measurement.

The pattern of variable dispersion observed in the CC deviates from the general assumption that it is a uniformly coherent fibre bundle of axons running parallel to one another. The observation of higher dispersion on the midline implies a reorientation of fibre trajectories at this level. The functional significance of this anatomical peculiarity, if any, is unclear. One appealing hypothesis is that fibres change direction at the midline to connect to heterotopic areas. Alternatively, it could be an epiphenomenon of callosal development. Several glial structures near the midline play an important role in the formation of the CC (Paul et al., 2007, Ren et al., 2006, Shu et al., 2003) and dispersion may be a remnant of the development of the CC.

Although the dispersion profiles measured with the different modalities showed a strong correlation (Fig. 12), PLI yielded lower ODI values than dMRI and histology. The spatial resolution of the PLI images may be the source of this discrepancy. At 4 µm/pix and a slice thickness of 60 µm, the orientation estimate of each PLI-pixel produces a single value that is the average of several dozens of myelinated axons within that neighbourhood. Hence, there is no sense in which the intrinsic PLI signal can be “broadened” by dispersion at the imaged resolution (Dohmen et al., 2015), unlike dMRI. Each myelin sheath acts as a wave retarder that induces a phase shift to the circularly polarized light. The net phase shift is simply a summation of all phase shifts induced by all myelin sheaths inside the pixel, creating the PLI signal. In contrast to dMRI, it is not possible to resolve within-pixel fibre configurations with PLI. Dispersion within a PLI pixel therefore cannot be quantified, and it is likely that PLI underestimates to some degree the dispersion values present in the full 3D tissue section.

Dispersion of two white matter compartments was investigated, myelinated axons and astrocytes, using the histology images. Diffusion in the extracellular space and in non-myelinated axons also contributes to dispersion, but these compartments were not evaluated in this study. However, the geometry of the extra-cellular space will be primarily defined by axons in white matter and similarly we think it is reasonable to assume that non-myelinated axons follow the same trajectory as myelinated axons. Thus, it was assumed that these compartments exhibit similar degrees of dispersion at a certain location as myelinated axons.

The primary type of astrocytes in white matter, fibrous astrocytes, have elongated processes (Kettenman and Ransom, 2013). It was hypothesized that this microstructure impedes diffusion and may contribute to diffusion anisotropy and dispersion. To test this hypothesis, we compared FODs based solely on myelin histology to an aggregate FOD including both myelin and astrocytes. Volume fractions for the myelin and astrocyte compartments were calculated based on area fractions in the histological images (Supplementary Figure S.3). The mapping between the area and volume fraction was based on a simulation that assumed a packing of randomly oriented fibres. Though this packing may not entirely hold true for myelinated axons and astrocytes, it provides some sense in how to convert the area fraction into a volume fraction. The average astrocyte volume fractions for our specimens ranged from f_A_ = 0.06 − 0.12, which agrees with the volume fractions found in literature, i.e. f_A_ = 0.1- 0.2 (Kettenman and Ransom, 2013). A closer resemblance between dMRI and histology was obtained when dispersion was only derived from the myelin FODs (Fig. 11). Because the dispersion estimated from astrocytes is much higher than that estimated from myelin, the aggregate FOD_MA_ yielded higher dispersion than the myelin FOD_M_ (Fig. 5). A regression analysis was performed on the dispersion profiles derived from FOD_M_ and FOD_A_ to consider whether any linear combination of the measured myelin and astrocyte FODs could improve (which could reflect, for example, a different volume fraction than that derived from our areal fraction simulations). This analysis yielded no contribution from astrocytes in addition to myelin to explain the dMRI dispersion profiles (data not shown). Taken together, these results suggest that, although exhibiting a similar spatial pattern of dispersion across the CC, astrocytic processes do not contribute significantly to the dMRI dispersion estimates.

**Fig S.4:**
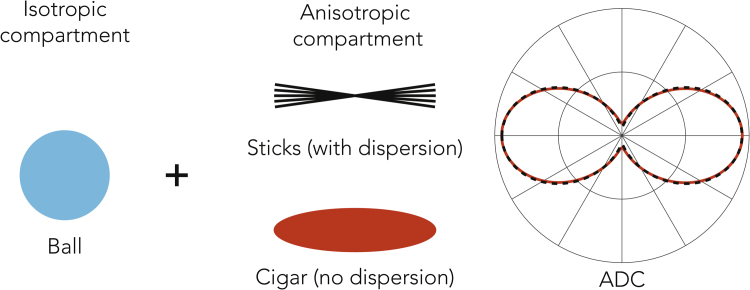
Forward simulation of a two-compartment model consisting of an isotropic and an anisotropic diffusion compartment. The FOD is shown as the apparent diffusion coefficient (ADC) in 2D. The FOD from a dispersing population of sticks is shown as the black dashed line. The sticks have no diffusion along the perpendicular direction. The simulation demonstrates that a geometry without dispersion, but with certain diffusion along the perpendicular direction, i.e. a cigar-like response function can produce a similar FOD (red solid line) as the dispersed sticks.

The present study employs a dMRI dispersion model that assumes a stick-like fibre response function which allows no diffusion perpendicular to it. Though the isotropic compartment should account for diffusion in the perpendicular direction, it is plausible that the stick implementation overestimates dispersion. Indeed, using a forward simulation it can be shown that a model with cigar-like response function, in which some perpendicular diffusion occurs, generates a very similar FOD to a model with dispersion and a stick-like response function (Supplementary Figure S.4). Accurate estimation of the response function could help improve microstructure models such as the one employed here, as well as spherical deconvolution techniques that aim to estimate the fODF from dMRI data. Currently, the response function is often estimated in the CC (Tax et al., 2014, Tournier et al., 2004). Our results demonstrate that dispersion in the CC cannot be neglected and is likely to have an effect on the estimated fibre response function and thus the fODF (Parker et al., 2013). Our framework could provide a way to calibrate the response function based on microscopy data. By matching the dispersion estimates from dMRI and histology, a mapping between the ground-truth fibre architecture and the dMRI representation may be obtained. Such a mapping would enable more accurate estimate of the fODF, which we aim to address in future work.

Finally it should be noted that this study considered one specific model (Sotiropoulos et al., 2012) to estimate dispersion from dMRI data. Recently, NODDI-Bingham (Tariq et al., 2016) was developed which generates very similar output parameters, as it also employs a Bingham distribution to characterize the fODF. It is beyond the scope of this study to compare these different dispersion models with each other, but it would certainly be of great interest whether other dMRI models provide a better match to the microscopy estimates. The data presented here may thus be a useful test-bed to further evaluate different models. Although not used in this study, the acquired dMRI protocol acquired two b-value shells, with an additional 120 gradient directions acquired at b = 2500 s/mm^2^. Data presented in this study is publically available at http://www.fmrib.ox.ac.uk/DigitalBrainBank, allowing other researchers to use it to evaluate their models.

## Conclusion

This work compared fibre orientation dispersion values derived from dMRI data using a two-compartment model with microscopic correlates. In terms of average dispersion profiles, high correlation was found between the dMRI and microscopy, both across regions and within the CC. Though correlative patterns of dispersion were found between the modalities, the absolute dispersion values were different, with the highest values reported for histology, followed by dMRI and lowest for PLI. A separate analysis suggests that astrocytes do not significantly contribute to the dispersion estimates in dMRI. Beyond an evaluation of dispersion estimates from dMRI, this kind of data may serve as a useful tool to study the mapping between ground-truth microstructure and its representation by dMRI. In particular, a microscopy informed estimation of the fibre response function will be addressed in future work.

## Figures and Tables

**Fig S.2:**
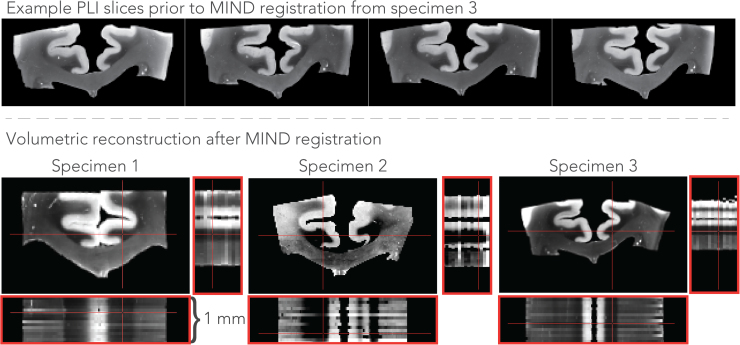
Results of stacking the PLI slices after registration with the MIND algorithm. The top row depicts example slices prior to registration to illustrate the deformations present between them. Each slice experiences different deformations that requires correction before slice stacking. The bottom row depicts the orthogonal views (red panels) of volumetric reconstruction after registration with the MIND algorithm. Note the alignment of the grey-white matter border across the different slices.

## Appendix A Dispersion model

The fibre orientation dispersion inside a voxel was estimated with a parametric two-compartment model (Sotiropoulos et al., 2012). In the case of perfectly aligned axons, the model can be simplified to a fully isotropic compartment and a stick-like compartment with diffusion completely restricted to be along the orientation of the stick (Behrens et al., 2003):

(A.1) $$S_{k}=S_{0}\left[\left(1-f\right)e^{-b_{k}d}\;+\;fe^{{-b_{k}d\left(g_{k}^{T}v\right)}^{2}}\right]$$

Here, S_k_ is a diffusion measurement of the k^th^ gradient along direction **g**_k_ with b-value b_k_, S_0_ a measurement with zero diffusion weighting and d a scalar diffusion coefficient. The volume fraction of the anisotropic compartment is given by f ∈ [0,1] that has orientation v=[sinθcosφ,sinθsinφ,cosθ] , φ ∈ [0-2π] and θ ∈ [0-π].

An extension to this model was implemented to capture orientation dispersion of fibres within a voxel. Instead of modelling fibres with a single orientation, they are assumed to form a population of sticks spatially distributed according to a distribution of orientations H(θ,φ):

(A.2) $$S_{k}=S_{0}[\left(1-f\right)e^{-b_{k}d}\;+\;f\int_{0}^{2\pi }\int_{0}^{\pi }H\left(\theta ,\varphi \right)e^{{-b_{k}d\left(g_{k}^{T}v\right)}^{2}}\sin\;\theta \;d\theta d\varphi ]$$

Here, H(**v**) is a Bingham distribution F_B_(**v**;**B**). Its probability density function is given by:

(A.3) $$F_{B}\left(v;B\right)=\frac{1}{C_{B}}e^{v^{T}\mathrm{Bv}}\mathrm{with}C_{B}=4\pi_{1}F_{1}(\frac{1}{2};\frac{3}{2};B)$$

and _1_F_1_(α; β; **Z**) is the confluent hypergeometric function of the first kind with matrix argument Z (approximated as described in (Sotiropoulos et al., 2012)). The Bingham matrix **B** is 3 × 3 symmetric matrix represented as **B** = **R**^T^**B**_diag_**R**, with R a rotation matrix and B_diag_ a diagonal matrix with two non-zero eigenvalues, k_1_ and k_2_ representing the dispersion.

(A.4) $$B_{\mathrm{diag}}=-\left[\begin{array}{ccc} k_{1} & 0 & 0\\ 0 & k_{2} & 0\\ 0 & 0 & 0 \end{array}\right]$$

The eigenvalues are reciprocally related to dispersion, i.e. the larger the eigenvalues the less dispersion is present. In the case of isotropic fanning k_1_ = k_2_ and a special form of the Bingham distribution appears, also known as the Watson distribution. For k_1_ > k_2_ the dispersion profile has an anisotropic shape.

## Appendix B 2D dispersion

For comparison with microscopy, dMRI dispersion was extracted from a plane parallel to the imaging plane of the microscopy data. The microscopy imaging plane was roughly perpendicular to the z-axis of the dMRI data. Let **x** be a 3D vector and the orientation distribution function ODF defined as **x** → ODF(**x**/|**x**|) obtained using the dMRI dispersion model described in Appendix A. The ODF(**x**) is projected onto a 2D plane and becomes pODF(**x**) = ODF(**P.x** / |**P.x**|), where **P** is a projector defined as:

$$P=\left[\begin{array}{ccc} 1 & 0 & 0\\ 0 & 1 & 0\\ 0 & 0 & 0 \end{array}\right]$$

The resulting pODF(**x**) was normalised to obtain a pdf to which a Bingham distribution was fitted (Riedel, 2015). Fitting the Bingham distribution to 2D samples theoretically sets k_1_ → ∞, leading to a single meaningful dispersion axis, since pODF(**x**) is a delta function in the third dimension. Hence, the eigenvalue representing the only non-zero dispersion, referred to as k_2D_, quantifies dispersion that is parallel to the microscopy plane.

## Appendix C PLI signal analysis

The light intensity for a birefringent specimen inside a PLI microscopy setup is described with the Jones calculus (Jones 1941),

(C.1) $$I\left(\rho \right)=\frac{I_{0}}{2}[1+\sin\left(2\rho -2\varphi \right)\sin\delta ]$$

where,

(C.2) $$\delta \approx 2\pi \frac{d\;∙\;\Delta n}{\lambda }\cos^{2}\alpha$$

Here, I_0_ is the average light intensity (transmittance), ρ the polarizer orientation and φ stands for the in-plane orientation of the fibre. The induced phase retardance (|sin δ|) is determined by the thickness of the specimen (d), the birefringence of the specimen (Δn), the wavelength of the light (λ) and the inclination angle of the myelin sheath (α).

## Appendix D Structure tensor analysis for histological image

To estimate local orientation of fibres, structure tensor analysis (Bigun and Granlund, 1987) was applied to the histological images. A pixel-wise method was adopted as described previously (Budde and Frank, 2012a, Rezakhaniha et al., 2012). For each pixel, a local structure tensor, a positive 2 × 2 matrix, J was defined

(D.1) $$J=\left[\begin{array}{cc} <I_{x},I_{x}{>}_{w} & <I_{x},I_{y}{>}_{w}\\ <I_{x},I_{y}{>}_{w} & <I_{y},I_{y}{>}_{w} \end{array}\right]$$

where I_x_ and I_y_ are the directional derivatives of an image I(x,y) along directions x and y, respectively. The inner product of the derivatives is convolved with a Gaussian weighting function, w, that defines the area of interest specified by a width (σ). This is inner product is defined as,

(D.2) $$<g,h{>}_{w}=\iint_{R^{2}}w\left(x,y\right)g\left(x,y\right)h\left(x,y\right)dxdy$$

where g(x,y) and h(x,y) are two arbitrary images. The predominant fibre orientation is given by θ,

(D.3) $$\theta =\frac{1}{2}\arctan(2\frac{<I_{x},I_{y}{>}_{w}}{<I_{y},I_{y}{>}_{w}-<I_{x},I_{x}{>}_{w}})$$
